# Supplementary material for: SNc Nuclease Genes AtCAN1 and AtCAN2 Are Expressed in Programmed Cell Death and Endoreduplicating Tissues in Arabidopsis thaliana
Source: Int J Mol Sci. 2026 Jun 16;27(12):5408. doi: 10.3390/ijms27125408 (PMC13300488; doi:10.3390/ijms27125408)
Supplement: Supplementary file 1 [file ijms-27-05408-s001.zip › ijms-4261529-supplementary.pdf]

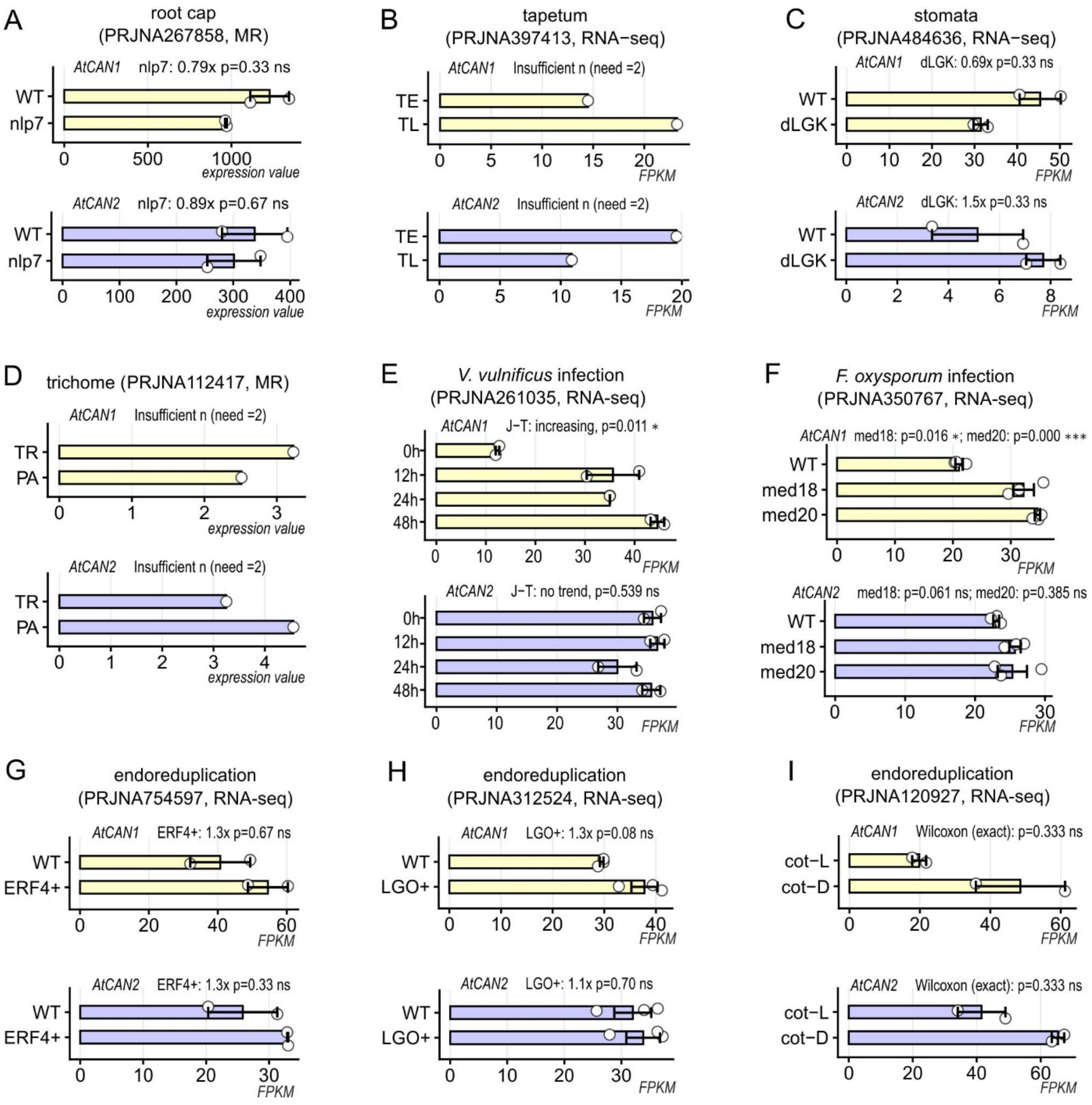

Figure S1. Transcriptome-based analysis of *AtCAN1* and *AtCAN2* gene expression profiles complements the information presented in Figures 7–9. The data shown relate to correlations described in this study between the expression of these two genes and different forms of programmed cell death (PCD), responses to pathogen-related stimuli, and endoreduplication. In each panel, two graphs show the expression of the genes encoding AtCAN1 (above, yellow) and AtCAN2 (below, purple). All methodological and technical details, including statistical test abbreviations, are as described in Figure 7. (A) Effect of the dNLP7 mutation on the expression of the analyzed genes. (B) The level of expression of the studied genes in tapetal cells at early (TE) and late (TL) stages of development, leading to programmed cell death (PCD). (C) Expression of *AtCAN1* and *AtCAN2* in wild-type (WT) plants versus *A. thaliana* FAMALGK mutants (dLGK) with impaired guard cell identity. *AtCAN1* shows a non-significant reduction in expression; *AtCAN2* shows the opposite change. (D) Comparison of the expression levels of the analyzed nuclease genes in trichomes (TR) and pavement epidermal cells (PA). (E) Expression levels of the studied genes from 0 (0h) to 48 h (48h) post-infection with *Vibrio vulnificus*. (F) Expression levels of the studied genes in WT and in MED18 (med18) or MED20 (med20) mutant plants upon infection with the pathogenic fungus *Fusarium oxysporum*. (G) Expression levels of the analyzed genes in wild-type (WT) plants and in plants with elevated endoreduplication induced by ERF4 overexpression (ERF4+). (H) Expression levels of the analyzed genes in wild-type (WT) plants and plants with increased endoreduplication induced by LGO overexpression (LGO+). (I) Expression levels of the analyzed genes in cotyledons induced to undergo endoreduplication through growth in darkness (cot-D) relative to light-grown control cotyledons (cot-L).
